# Patient Acceptance of Noninvasive and Invasive Coronary Angiography

**DOI:** 10.1371/journal.pone.0000246

**Published:** 2007-02-28

**Authors:** Eva Schönenberger, Dirk Schnapauff, Florian Teige, Michael Laule, Bernd Hamm, Marc Dewey

**Affiliations:** 1 Department of Medicine, Medizinische Hochschule Hannover, Hannover, Germany; 2 Department of Radiology, Charité, Humboldt-Universität zu Berlin, Berlin, Germany; 3 Department of Cardiology, Charité, Humboldt-Universität zu Berlin, Berlin, Germany; Innsbruck Medical University, Austria

## Abstract

**Background:**

Noninvasive angiography using multislice computed tomography (MSCT) is superior to magnetic resonance imaging (MRI) for detection of coronary stenoses. We compared patient acceptance of these two noninvasive diagnostic tests and invasive conventional coronary angiography (Angio).

**Methods and Findings:**

A total of 111 consecutive patients with suspected coronary artery disease underwent MSCT, MRI, and Angio. Subsequently, patient acceptance of the three tests was evaluated with questionnaires in all patients. The main acceptance variables were preparation and information prior to the test, degree of concern, comfort, degree of helplessness, pain (on visual analog scales), willingness to undergo the test again, and overall satisfaction. Preparation for each test was not rated significantly differently, whereas patients were significantly more concerned about Angio than the two noninvasive tests (p<0.001). No pain during MSCT, MRI, and Angio as assessed on visual analog scales (0 to 100) was reported by 99, 93, and 31 patients, respectively. Among the 82 patients who felt pain during at least one procedure, both CT (0.9±4.5) and MRI (5.2±16.6) were significantly less painful than Angio (24.6±23.4, both p<0.001). MSCT was considered significantly more comfortable (1.49±0.64) than MRI (1.75±0.81, p<0.001). In both the no-revascularization (55 patients) and the revascularization group (56 patients), the majority of the patients (73 and 71%) would prefer MSCT to MRI and Angio for future imaging of the coronary arteries. None of the patients indicated to be unwilling to undergo MSCT again. The major advantages patients attributed to MSCT were its fast, uncomplicated, noninvasive, and painless nature.

**Conclusions:**

Noninvasive coronary angiography with MSCT is considered more comfortable than MRI and both MSCT and MRI are less painful than Angio. Patient preference for MSCT might tip the scales in favor of this test provided that the diagnostic accuracy of MSCT can be shown to be high enough for clinical application.

## Introduction

Coronary artery disease is affecting over 13 million people in the United States [1], [2]. Invasive conventional coronary angiography (Angio) is the gold standard for detection of this disease; however, it is invasive and carries a risk of 1.7% of relevant complications [3]. Noninvasive coronary angiography could overcome these risks and has been shown to be feasible with multislice computed tomography (MSCT, also known as multidetector row computed tomography, MDCT) [4]–[11] and magnetic resonance imaging (MRI) [12], [13]. MSCT requires intravenous administration of an iodinated contrast agent and exposes our patients to ionizing radiation while MRI is limited by the narrow bore which sometimes causes a feeling of confinement and claustrophobia. A recent meta-analysis has found MSCT to be significantly more accurate than MRI for detection of coronary stenoses [14]. To achieve widespread application, however, a new diagnostic test must also be highly accepted by patients. There is no data available in regards to the acceptance of the diagnostic tests for coronary artery stenosis detection. Thus, we prospectively compared patient acceptance of the two new noninvasive tests (MSCT and MRI) with that of the invasive reference standard – conventional coronary angiography (Angio) – in a consecutive cohort of 111 patients with suspected coronary artery disease as an ancillary study of an investigator-initiated study of noninvasive coronary angiography [15]. The results show that noninvasive coronary angiography with MSCT is considered more comfortable than MRI and both noninvasive tests are less painful than Angio. Independent of a subsequent coronary revascularization, the majority (over 70%) of the patients favors MSCT for future imaging of the coronary arteries.

## Methods

### Study Population

A total of 111 consecutive patients (28 women, mean age 63±8 years) with suspected coronary disease and without contraindications to MSCT, MRI, and Angio were prospectively included and underwent all three tests as part of the protocol of an investigator-initiated study on noninvasive coronary angiography [15]. Subsequently, patient acceptance was evaluated with a questionnaire (Text S1) in all patients. None of the patients had undergone any of the three tests prior to this study. The institutional review board and the Federal Department for Radiation Protection approved the study. All patients gave written informed consent and the procedures were performed according to the Declaration of Helsinki.

### MSCT Protocol

Scanning was performed on an MSCT scanner with 16×0.5 mm detector collimation (Aquilion 16, Toshiba Medical Systems, Otawara, Japan) as described [15] with retrospective ECG-gating, multisegment reconstruction, and an average image reconstruction interval of 147±36 ms [9], [16]. Sublingual nitroglycerine was administered to increase coronary artery diameters [17]. A mean volume of 108.2±10.9 mL of a nonionic, iso-osmolar contrast agent (iodixanol, 320 mg of iodine per mL, Visipaque, GE-Healthcare Biosciences, Buckinghamshire, United Kingdom) [18] was injected intravenously at a rate of 3.5 mL/s. Radiation exposure was estimated to be 12.2±1.4 mSv [19]. Patients with contraindications to MSCT (e.g. renal failure, allergy to iodinated contrast agents) were not included in this ancillary study on patient acceptance.

### MRI Protocol

Imaging was performed on a 1.5-T MRI scanner (Magnetom Sonata, Siemens Medical Solutions, Erlangen, Germany) using a dedicated cardiac 12-element phased-array coil and a high-performance gradient subsystem (maximum amplitude of 40 mT/m and a minimum rise time of 200 µs) as described [15]. A noncontrast balanced three-dimensional steady-state free precession sequence [20] was used to image the coronary arteries in volumes targeted along the left and right coronary artery system [12]. Images were acquired with the patient lying supine during free breathing or breath-hold as described [20]. Patients with contraindications to MRI (e.g. claustrophobia, pacemakers) were not included in this study on patient acceptance. Three of the patients included in the study prematurely terminated the examination due to claustrophia at least 5 min after beginning of the MRI and were not excluded from the study according to the STARD statement [21]. Ear protection was given to all patients in the form of headsets which also served to transmit the breathing instructions. Additional blankets for thermal comfort were available for the MR examination and were provided if desired by patients.

### Angio Protocol

All of the 111 patients underwent conventional angiography using standard techniques (Integris 3000, Philips Medical Systems, Best, the Netherlands) with the transfemoral approach and administration of an average amount of 95.9±22.0 mL of an iodinated contrast agent. Local anesthesia at the puncture site was performed using 20 mL of 1% lidocaine. A pressure dressing at the puncture site was applied for 6 hours in each patient after Angio. In patients who underwent percutaneous coronary intervention, sheath removal was performed with the assistance of a mechanical compression device (FemoStop) [22]. Altogether, the patients had to lie flat after the procedure for 12 hours.

### Questionnaire Design and Distribution

Prior to the diagnostic tests, the patients were informed about the nature and the purpose of the questionnaire (Text S1) in order to allow them to thoroughly register all important perceptions during the tests. Since this study was a sub-study of an investigator-initiated trial of the diagnostic performance of the two noninvasive tests no information in regards to diagnostic accuracy could be given to the patients. Patients were also asked to only assess their own experiences and preferences and try not to let themselves be influenced by the expected diagnostic value of the two noninvasive tests. Of course in clinical practice the decision to perform a diagnostic test is complex and is influenced by both its value and the patient or physician preferences. Therefore, in the present study we aimed at isolating the patients' views from the anticipated diagnostic accuracy to obtained unbiased results in this regard. MSCT and MRI were always performed prior to Angio with a median of 1 day between noninvasive and invasive coronary angiography. The first test performed was MSCT in 58 patients (52%) and MRI in 53 patients (48%) patients. The patients were instructed to fill out the questionnaire one day after all tests were completed. One of the investigators was available in person at the time of completion of the questionnaire to resolve any issues regarding certain questions. The main variables of patient acceptance for all three tests within the questionnaire were preparation and information prior to the test, degree of concern prior to the test, comfort during the test, degree of helplessness, and overall satisfaction. The variables were assessed using 5-point Likert scales. In addition, maximum subjective pain levels during all tests were recorded on horizontal marked visual analog scales (0 to 100 arbitrary units). The patients were also asked which of the tests they would prefer for future imaging of the coronary arteries and whether they would be willing to undergo the tests again. Open-ended questions allowed the patients to report any advantages and disadvantages of the tests as judged by them.

### Statistical Analysis

All data are expressed as mean±SD except those presented as frequencies. Wilcoxon's test for paired samples was applied to identify differences between the main variables of patient acceptance for all three tests. A contingency analysis with a χ^2^ test (if at least five cases were present in a single cell in a 2-by-2 table) or Fisher exact test (for less than five cases per cell) was used to compare both the willingness to undergo the tests again and the preference for one test between MSCT, MRI, and Angio. The paired t-test was used to identify differences in subjective pain assessed with visual analog scales and the duration of the three tests. Altogether 25 statistical tests were performed and consequently, adjustment for multiple measurements (Bonferroni) was used to reduce the probability of making a type-I error. Thus, not the commonly used p value <0.05, but a p value <0.002 was considered statistically significant. Sample size calculation for the main study was based on the aim to compare per-patient diagnostic accuracy of MSCT and MRI and to demonstrate that the negative predictive value of MSCT is greater than 90% [15]. No separate power analysis was performed for this ancillary study of patient acceptance. Statistical analyses were conducted using SPSS version 12.0.

## Results

### Pain on Visual Analog Scales

**Figure 1:**
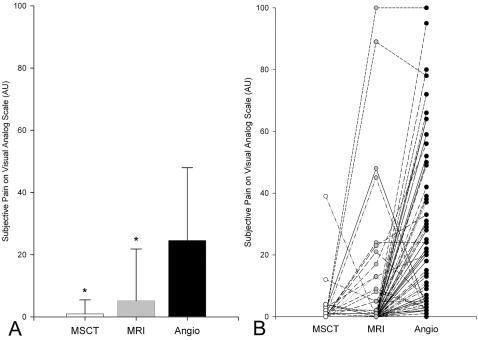
Average (+SD) subjective pain as assessed with visual analog scales during all three tests (*A*) and corresponding intraindividual comparisons of pain (*B*) among the 82 patients who indicated pain during at least one procedure. ^*^ p<0.001 compared with Angio using the paired t-test. MSCT = multislice computed tomography; MRI = magnetic resonance imaging; Angio = conventional coronary angiography.

All of the 111 patients underwent all three tests and completed the questionnaire entirely (100% response rate). No pain during MSCT, MRI, and Angio on visual analog scales was reported by 99, 93, and 31 patients, respectively. Twenty-nine patients indicated no pain during any test. Among the 82 patients (74%) who felt pain during at least one procedure, both MSCT (0.9±4.5) and MRI (5.2±16.6) were experienced as significantly less painful than Angio (24.6±23.4, p<0.001, Figure 1A). Pain values were not significantly different between patients who underwent subsequent percutaneous coronary intervention and those who did not. The intraindividual comparison of subjective pain values presented in Figure 1B shows that one patient reported worst imaginable pain (100 arbitrary units) during MRI and Angio. This patient suffered severe back pain and headache as a result of having to lie flat for a long time during and after Angio and during MRI. Two other patients reported pain of equal severity in two of the tests. In 70 of the 82 patients (85%) who felt pain during at least one test, most pain was reported to have occurred during Angio. Two (2%) and 7 (9%) patients felt most pain during MSCT and MRI, respectively (Figure 1B). The maximum reported subjective pain reported for MSCT was 39 arbitrary units on the visual analog scale in a patient who underwent a complex venipuncture procedure.

### Patient Acceptance

The results for the different variables of patient acceptance are shown in Table 1. MSCT was considered significantly more comfortable than MRI (p<0.001), and the patients indicated a significantly lower degree of helplessness during MSCT than during Angio (p<0.001). The degrees for preparation, comfort, helplessness, and overall satisfaction were in a good to very good range for all three tests (Table 1). Preparation and information for each test was not evaluated significantly differently, whereas patients were significantly more concerned prior to the tests about conventional coronary angiography than about either of the two noninvasive tests (p<0.001). Overall satisfaction was higher for MSCT than for MRI and Angio, but the difference was not significant after adjustment for multiple measurements (p = 0.003 and p = 0.019, respectively, Table 1).

**Table 1:** Results of Patient Acceptance for all Three Tests

	MSCT	MRI	Angio
Preparation and information prior to the test*	1.27±0.52	1.35±0.64	1.48±0.72
Degree of concern prior to the test†	1.51±0.85‡	1.64±0.93‡	2.75±1.23
Comfort during the test*	1.49±0.64§	1.75±0.81	1.54±0.68
Degree of helplessness†	1.19±0.48‡	1.39±0.89	1.52±0.86
Overall satisfaction*	1.32±0.51	1.58±0.89	1.46±0.61

* evaluated on a five-point scale (range: 1 = very good to 5 = poor)

† evaluated on a five-point scale (range: 1 = none to 5 = very high)

‡ p<0.001 compared with conventional coronary angiography using Wilcoxon's test for paired samples

§ p<0.001 compared with MRI using Wilcoxon's test for paired samples

MSCT = multislice computed tomography; MRI = magnetic resonance imaging; Angio = conventional coronary angiography.

### Overall Preference and Revascularization

**Figure 2:**
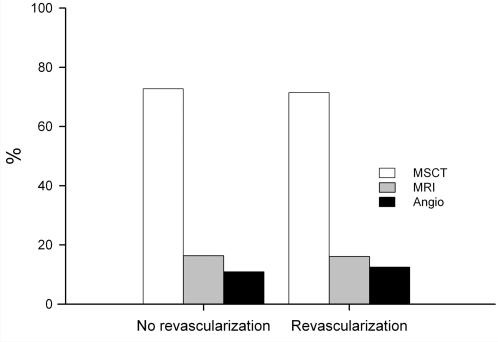
Comparison of the preference for one of the three diagnostic tests between the 55 patients who received no coronary revascularization (No revascularization) and the 56 patients who underwent subsequent percutaneous or surgical revascularization (Revascularization). Response alternatives were MSCT, MRI, and Angio. The preference for MSCT was only slightly and not significantly reduced in the “Revascularization” group (71%) as compared to the “No revascularization” group (73%), while 12.5% and 11% of the patients in these two groups preferred Angio, respectively.

Overall, 80 of the 111 patients (72%) preferred MSCT, whereas 18 (16%) patients preferred MRI and 13 (12%) patients Angio (Table 2). This overall preference for MSCT was significantly higher than that for MRI and Angio (p<0.001), whereas the difference between MRI and Angio was not significant using the χ^2^ test (p = 0.33, Table 2). Of the 111 patients included, 55 received no coronary intervention and 56 patients underwent subsequent percutaneous (36 patients) or surgical (20 patients) revascularization. In both the no-revascularization and the revascularization group, the vast majority of the patients (73 and 71%) would prefer MSCT to MRI and Angio for future imaging of the coronary arteries (Figure 2). There was no significant difference between these groups in the preference for MSCT.

**Table 2:** Results of Overall Patient Preference for the Three Tests

	MSCT	MRI	Angio
Preferred test	80 (72%)	18 (16%)	13 (12%)
Not the preferred test	31 (28%)	93 (84%)	98 (88%)

Overall patient preference was significantly higher for MSCT compared with MRI and Angio (both: p<0.001). A detailed comparison of the preferences for each of the three tests for patients with and without subsequent coronary revascularization is given in Figure 2.

### Future Examinations

**Figure 3:**
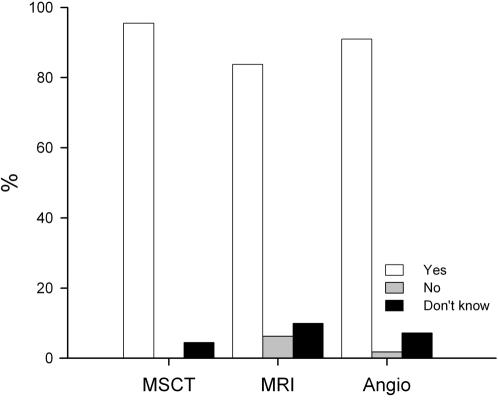
Willingness of the 111 patients to undergo the tests again.

Regarding their willingness to undergo the tests again, none of the patients declined a future examination with MSCT, whereas 7 and 2 patients, respectively, indicated that they would dislike another MRI and Angio examination (Figure 3). The difference between MSCT and MRI regarding the patients' willingness to repeat the test, was not significant after adjustment for multiple measurements (p = 0.006). Five, 11, and 8 patients, respectively, did not know whether they would undergo MSCT, MRI, and Angio again (Figure 3).

### Open-ended Questions

Advantages and disadvantages of the tests as seen by the patients are summarized in Table 3. The most important advantages of MSCT from the patients' perspective were the short duration and the uncomplicated, noninvasive, and painless nature of this test. The advantages of MRI were the absence of radiation, noninvasiveness, and painlessness. The patients appreciated the therapeutic capabilities and high accuracy of Angio, and also the chance to see the images during this examination. Only 37 disadvantages were suggested by 34 patients for MSCT, among them radiation exposure and contrast agent administration as the most important ones. In contrast, for MRI and Angio, 105 and 112 disadvantages were suggested by 73 and 75 patients, respectively. The long examination, the sensation of confinement, and noise were the most important disadvantages of MRI, while Angio was considered to be limited mostly by the need to lie flat after the procedure, the pressure dressing, the invasiveness, and pain (Table 3).

**Table 3:** Advantages and Disadvantages of the Three Tests as Suggested by the Patients*

	MSCT	MRI	Angio
Advantages (no.; %)	**n = 104** given by **79 patients**	**n = 42** given by **38 patients**	**n = 65** given by **59 patients**
	Fast (63; 61%)	No radiation (13, 31%)	Therapy possible (33; 51%)
	Uncomplicated (13; 13%)	Noninvasive (11; 26%)	Highest accuracy (16; 25%)
	Painless (11; 11%)	Painless (7; 17%)	Images during the examination (8, 12%)
	Noninvasive (7; 7%)	Uncomplicated (3; 7%)	Faster information regarding findings (3; 5%)
	No confinement (4; 4%)	Fast (2; 5%)	Fast (2; 3%)
	Noncardiac findings, Low risk, Outpatient setting, Silent, No fear, Comfortable, (each 1; 1%)	Noncardiac findings, Low risk, Outpatient setting, No contrast agent, Images immediately available, Active cooperation of the patient (each 1; 2%)	Painless (2; 3%)
			Not alone (1; 2%)
Disadvantages (no.; %)	**n = 37** given by **34 patients**	**n = 105** given by **73 patients**	**n = 112** given by **75 patients**
	Radiation (23; 62%)	Long examination and long lying flat (43; 41%)	Long lying flat after the procedure (35; 31%)
	Contrast agent (7; 19%)	Confinement (34; 32%)	Invasive (17; 15%)
	No therapy (3; 8%)	Noise (9; 9%)	Pressure dressing (15; 13%)
	Long breathhold (3; 8%)	Long and frequent breathholds (7; 7%)	Pain (14; 13%)
	No online-images (1; 3%)	Strenuous lying flat in unchanged position (3; 3%)	Time-consuming preparation and aftercare (8; 7%)
		Great strain (3; 3%)	Possible adverse events (8; 7%)
		Being alone (2; 2%)	Inpatient setting (3; 3%)
		No therapy, Active cooperation of the patient, Fan, Felt cold (each 1; 1%)	Radiation (2; 2%)
			Groin hematoma (2; 2%)
			Not possible to use the restroom (2; 2%)
			Contrast agent, Duration, More expensive, Narrow table, Sensation of the catheter in the heart, Psychological stress (each 1; 1%)

* multiple suggestions per patient possible. Percentages in brackets are in relation to the number of advantages/disadvantages given for this respective test.

## Discussion

In this study on patient acceptance of different diagnostic tests for coronary angiography, MSCT was considered more comfortable than MRI and both noninvasive tests were less painful than Angio. Independent of a subsequent coronary revascularization, the majority of the patients favored MSCT for future imaging of the coronary arteries. From the patients' perspective the main reasons for the high acceptance of MSCT are: it is uncomplicated, noninvasive, painless, and fast (Table 3). This subjective assessment is corroborated by the comparison of the total duration of the different tests, which shows that MSCT was significantly faster (17.4±5.9 min) than both MRI (58.4±11.4 min, p<0.001) and Angio (57.9±16.7 min, excluding time necessary for interventions, p<0.001).

### Clinical Considerations and Limitations

**Figure 4:**
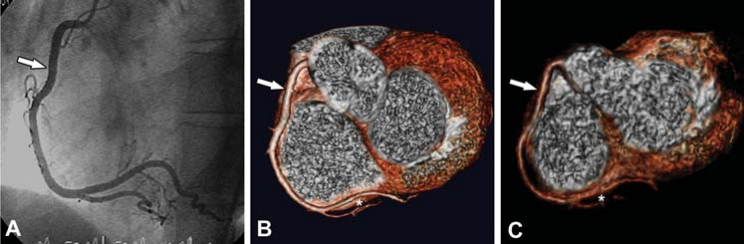
Angio (*A*) and noninvasive coronary angiography using MSCT (*B*) and MRI (*C*) all demonstrate absence of significant stenoses in the right coronary artery (arrow) in a 45-year-old female patient with atypical angina pectoris. Note that MSCT due to higher spatial resolution allows better delineation of the distal segments of the right coronary artery than MRI (asterisks).

To rule out coronary artery stenosis reliably is the foremost aim of noninvasive coronary angiography using either MSCT or MRI (Figure 4). From a clinical perspective the current study is of importance, since a new diagnostic test will not gain widespread clinical application before its acceptance by patients has been proven. It should be noted, however, that not only patient preferences but also the preference of the physician determines which tests a patient is send to. Hence, a patient is very likely to follow the initial recommendation of his general practitioner and then fail to show up for an appointment if the test is said to be rather uncomfortable. This “no show” rate might therefore be lowered if tests can be offered that are more comfortable and less painful. As a result, there will be more effective utilization of the imaging time slots of the new diagnostic test. We also believe that the present results should and will strongly influence physicians' recommendations of diagnostic tests. Thus, our results are of importance not only to patients and patient associations but also to the medical profession. To the best of our knowledge, the present study shows for the first time in a detailed analysis the different aspects of patient preferences for the diagnostic tests available for coronary angiography.

As shown in investigations on virtual colonoscopy with MSCT [23]–[25], cholangiopancreaticography [26], and carotid angiography [27] with MRI, those noninvasive tests have the potential to become more widely accepted by patients than conventional interventional approaches. This is mostly due to the fact that noninvasive approaches are less likely to be associated with adverse reactions and significant pain. The present study extends the knowledge of these studies to the area of coronary angiography. In contrast to other investigations on patient acceptance [23]–[27], we also compared two noninvasive tests against each other, which revealed that commonly cited drawbacks of MRI (sensation of confinement [34 patients], rather lengthy test [43 patients], and noise [9 patients]) led to significantly lower patient comfort than achieved with MSCT. MR scanners with less confinement and noise and a patient-centered design are very likely to improve comfort and reduce claustrophobia during MR examinations. This is of relevance, because new drug developments (blood-pool contrast agents) are likely to improve the diagnostic accuracy of MRI for detection of coronary stenoses [28], [29]. This new MRI approach might close the gap to MSCT in diagnostic accuracy. However, despite these promising results, the inconvenient circumstances of MRI examinations (narrow bore) need to be further alleviated by the development of more patient-friendly MRI-scanner design and technology before MRI can become a clinically useful test for noninvasive coronary angiography. This also underlines the fact that the present analysis of patient acceptance of coronary angiography is merely a snapshot in time and further improvements in technology might not only improve the diagnostic performance but also the acceptance of any of the three tests. The rather long time the patients had to lie flat after Angio during the present study might have contributed to the assessment of this diagnostic test. However, a mechanical compression device that increases acceptance by patients [22] was used for sheath removal in patients who underwent percutaneous coronary intervention.

Interestingly, only 1 patient complained about the use of contrast agents for Angio, whereas 7 patients mentioned the contrast agent as a drawback of MSCT. This difference in patient perception may be explained by the fact that the mode of contrast administration is different for the two examinations (multiple small injections into the coronary arteries for Angio and a single large intravenous injection for MSCT). Thus, further reducing the contrast agent amount required (as possible with 64-slice CT) might make MSCT even more attractive. However, there might be another explanation for the differences seen in contrast agent acceptance between MSCT and Angio. Both tests involve radiation exposure but this is again more often mentioned as a drawback of MSCT (23 patients) than of Angio (2 patients). Thus, the observed differences in the perception of MSCT and Angio might be due to the fact that patients concentrate on the main disadvantages of each test, which, in the case of Angio, are invasiveness [17 patients], pressure dressing [15 patients], pain [14 patients], and having to lie flat for a long time afterwards [35 patients], and thus ignore the fact that Angio involves contrast administration and radiation exposure as well. Interesting drawbacks of MRI as cited by our patients are the long examination time (43 patients) and the frequent long breathholds (7 patients). These inconveniences might be overcome in the near future with the use of new faster MR sequences such as parallel imaging approaches [30]. This holds also true for other complaints reported for MR coronary angiography like “great strain” and “strenuous lying flat in unchanged position” (each 3 patients). In our in-patient research setting it was impossible to have a friend or relative present in the MR scanner room if desired (disadvantage mentioned by 2 patients). In clinical practice, however, it is possible to provide for the presence of a close person if it is known that this would make the MR examination more comfortable for some patients. The major concern expressed by our patients about MSCT was the use of ionizing radiation exposure (23 patients). Conflicting results are reported with regard to the effective radiation dose of 16-slice CT coronary angiography in direct comparison to conventional coronary angiography – Coles et al. found significantly higher doses for CT (including calcium scoring) in a study of 91 patients (14.7±2.2 mSv vs. 5.6±3.6 mSv),[31] whereas we found no significant differences between both tests (12.3±1.4 mSv vs. 11.4±4.8 mSv) in a subgroup of 73 patients [15]. The radiation exposure might even increase with 64-slice CT because of a higher overranging effect and more scattered radiation. Thus, the main goal for MSCT coronary angiography in the near future must be to further reduce radiation exposure for instance by tube current modulation [32], [33] or prospectively triggered scanning using 256-slice CT [34] to reduce the cancer-inducing risk of this examination and thus make the test more acceptable to patients. Unlike noninvasive coronary angiography approaches, Angio allows performing subsequent coronary revascularization (mentioned by 33 patients) in the same session. No comparable therapeutic capability is in sight for MSCT and MRI in coronary artery disease. Despite the fact that patients were asked not to let themselves be influenced by the (expected) diagnostic value of the tests, 16 patients mentioned the high diagnostic accuracy of Angio as a relevant advantage of this test. Thus, the overall acceptance of Angio might have been positively biased by the fact that some patients did not strictly separate their own subjective preference from the diagnostic accuracy of the tests. The in-patient setting of Angio was considered a relevant drawback by 3 patients, to which our research setting in a large University hospital may also have contributed. This drawback can be overcome by conducting this test in private practice. With regard to its other drawbacks (Table 3), Angio differs from MSCT and MRI in that they will not be easily overcome by new developments.

Our study had some limitations including the single-center design and the inclusion of a small number (111) of patients. Also the order of the tests could have influenced patient preference since patients might be more prone to be dissatisfied with the one that came second because of waiting time. Patients were not randomized to undergo CT or MRI first but there was no relevant difference in the order of the tests in the present study (52% CT first vs. 48% MRI first). We did not compare patient acceptance of noninvasive cardiac stress tests (such as treadmill exercise), which might be considered more comfortable, with that of noninvasive and invasive coronary angiography. Strengths of the present study include the performance of three tests for coronary angiography in all patients, the consecutive inclusion of patients, the prospectively applied protocol of patient acceptance measurement, and the intention-to-diagnose design.

### Potential Clinical Role of MSCT and MRI

The potential clinical role and test performance of MSCT and MRI is subject of a separate report of the study [15], while this ancillary analysis was focused on patient acceptance. A recent meta-analysis has shown significantly higher accuracy for MSCT compared with MRI for detection of coronary stenoses [14] while our large head-to-head comparison has demonstrated a significantly higher per-patient sensitivity of MSCT compared with MRI [15]. Studies on the clinical utility of MSCT and MRI in determining the most suitable strategy for patient management have not been performed thus far and are highly desirable to thoroughly analyze which patients might benefit most from these noninvasive tests. CT coronary angiography might become a cost-effective approach to detection of coronary artery disease [35]. In our study the vast majority of patients would prefer MSCT over MRI and Angio for future diagnostic imaging of the coronary arteries. Also MSCT was significantly more comfortable than MRI and both noninvasive tests were less painful than Angio. Thus, from the patients' perspective, Angio could be replaced with MSCT in certain patient groups (e.g. ruling out stenoses in patients with a low-to-intermediate likelihood of coronary artery disease) [36]. Patient acceptance should also be analyzed in future studies using 64-slice CT [37]–[42]. It should be noted that just because patients prefer a new test does not necessarily mean that it is justified to perform this test in clinical practice since a test must also prove high diagnostic accuracy and reliability before its widespread application can be recommended. A recently published multicenter study on 16-slice CT coronary angiography for instance has shown important limitations of this technology in regards to diagnostic accuracy [43] and the widespread use of MSCT for the coronary arteries is still in question. However, patient acceptance needs to be considered a prerequisite for successful implementation into clinical practice and the intraindividual comparison demonstrated a clear preference for MSCT in our study.

The results of the present study suggest that patients prefer noninvasive coronary angiography over Angio because it is significantly less painful and MSCT coronary angiography is considered more comfortable than MRI. Thus, provided that the diagnostic accuracy of MSCT can be shown to be high enough for clinical application, patient preference for MSCT might tip the scales in favor of this test.

## Supporting Information

Text S1 Patient Acceptance Questionnaire. Main Variables of Patient Acceptance.
(0.09 MB PDF)

## References

[1] Cooper R, Cutler J, Desvigne-Nickens P, Fortmann SP, Friedman L (2000). Trends and disparities in coronary heart disease, stroke, and other cardiovascular diseases in the United States: findings of the national conference on cardiovascular disease prevention.. Circulation.

[2] American Heart Association (2004). Heart Disease and Stroke Statistics - Update.

[3] Noto TJ,, Johnson LW, Krone R, Weaver WF, Clark DA (1991). Cardiac catheterization 1990: a report of the Registry of the Society for Cardiac Angiography and Interventions (SCA&I).. Cathet Cardiovasc Diagn.

[4] Nieman K, Cademartiri F, Lemos PA, Raaijmakers R, Pattynama PM (2002). Reliable noninvasive coronary angiography with fast submillimeter multislice spiral computed tomography.. Circulation.

[5] Ropers D, Baum U, Pohle K, Anders K, Ulzheimer S (2003). Detection of coronary artery stenoses with thin-slice multi-detector row spiral computed tomography and multiplanar reconstruction.. Circulation.

[6] Mollet NR, Cademartiri F, Nieman K, Saia F, Lemos PA (2004). Multislice spiral computed tomography coronary angiography in patients with stable angina pectoris.. J Am Coll Cardiol.

[7] Martuscelli E, Romagnoli A, D'Eliseo A, Razzini C, Tomassini M (2004). Accuracy of thin-slice computed tomography in the detection of coronary stenoses.. Eur Heart J.

[8] Hoffmann MH, Shi H, Schmitz BL, Schmid FT, Lieberknecht M (2005). Noninvasive coronary angiography with multislice computed tomography.. Jama.

[9] Dewey M, Laule M, Krug L, Schnapauff D, Rogalla P (2004). Multisegment and halfscan reconstruction of 16-slice computed tomography for detection of coronary artery stenoses.. Invest Radiol.

[10] Schoepf UJ, Becker CR, Ohnesorge BM, Yucel EK (2004). CT of coronary artery disease.. Radiology.

[11] Schoenhagen P, Halliburton SS, Stillman AE, Kuzmiak SA, Nissen SE (2004). Noninvasive imaging of coronary arteries: current and future role of multi-detector row CT.. Radiology.

[12] Kim WY, Danias PG, Stuber M, Flamm SD, Plein S (2001). Coronary magnetic resonance angiography for the detection of coronary stenoses.. N Engl J Med.

[13] Bogaert J, Kuzo R, Dymarkowski S, Beckers R, Piessens J (2003). Coronary artery imaging with real-time navigator three-dimensional turbo-field-echo MR coronary angiography: initial experience.. Radiology.

[14] Schuijf JD, Bax JJ, Shaw LJ, de Roos A, Lamb HJ (2006). Meta-analysis of comparative diagnostic performance of magnetic resonance imaging and multislice computed tomography for noninvasive coronary angiography.. Am Heart J.

[15] Dewey M, Teige F, Schnapauff D, Laule M, Borges AC (2006). Noninvasive Detection of Coronary Artery Stenoses with Multislice Computed Tomography or Magnetic Resonance Imaging.. Ann Intern Med.

[16] Dewey M, Müller M, Teige F, Schnapauff D, Schink T (2006). Multisegment and halfscan reconstruction of 16-slice computed tomography for assessment of regional and global left ventricular myocardial function.. Invest Radiol.

[17] Dewey M, Hoffmann H, Hamm B (2006). Multislice CT coronary angiography: effect of sublingual nitroglycerine on the diameter of coronary arteries.. Fortschr Röntgenstr.

[18] Aspelin P, Aubry P, Fransson SG, Strasser R, Willenbrock R (2003). Nephrotoxic effects in high-risk patients undergoing angiography.. N Engl J Med.

[19] Stamm G, Nagel HD (2002). CT-expo–a novel program for dose evaluation in CT.. Röfo.

[20] Dewey M, Teige F, Schnapauff D, Laule M, Borges AC (2006). Combination of Free-breathing and Breath-hold Steady-state Free Precession Magnetic Resonance Angiography for Detection of Coronary Artery Stenoses.. J Magn Reson Imaging.

[21] Bossuyt PM, Reitsma JB, Bruns DE, Gatsonis CA, Glasziou PP (2003). Towards complete and accurate reporting of studies of diagnostic accuracy: The STARD Initiative.. Ann Intern Med.

[22] Stiebellehner L, Nikfardjan M, Diem K, Atteneder M, Stulnig T (2002). Manual compression versus mechanical compression device (FemoStop) after diagnostic coronary angiography with/without intervention.. Wien Klin Wochenschr.

[23] Svensson MH, Svensson E, Lasson A, Hellstrom M (2002). Patient acceptance of CT colonography and conventional colonoscopy: prospective comparative study in patients with or suspected of having colorectal disease.. Radiology.

[24] Lefere PA, Gryspeerdt SS, Dewyspelaere J, Baekelandt M, Van Holsbeeck BG (2002). Dietary fecal tagging as a cleansing method before CT colonography: initial results polyp detection and patient acceptance.. Radiology.

[25] van Gelder RE, Birnie E, Florie J, Schutter MP, Bartelsman JF (2004). CT colonography and colonoscopy: assessment of patient preference in a 5-week follow-up study.. Radiology.

[26] Menon K, Barkun AN, Romagnuolo J, Friedman G, Mehta SN (2001). Patient satisfaction after MRCP and ERCP.. Am J Gastroenterol.

[27] U-King-Im JM, Trivedi R, Cross J, Higgins N, Graves M (2004). Conventional digital subtraction x-ray angiography versus magnetic resonance angiography in the evaluation of carotid disease: patient satisfaction and preferences.. Clin Radiol.

[28] Taupitz M, Schnorr J, Wagner S, Kivelitz D, Rogalla P (2001). Coronary magnetic resonance angiography: experimental evaluation of the new rapid clearance blood pool contrast medium P792.. Magn Reson Med.

[29] Herborn CU, Barkhausen J, Paetsch I, Hunold P, Mahler M (2003). Coronary arteries: contrast-enhanced MR imaging with SH L 643A–experience in 12 volunteers.. Radiology.

[30] Niendorf T, Sodickson D (2006). [Acceleration of cardiovascular MRI using parallel imaging: basic principles, practical considerations, clinical applications and future directions].. Rofo.

[31] Coles DR, Smail MA, Negus IS, Wilde P, Oberhoff M (2006). Comparison of radiation doses from multislice computed tomography coronary angiography and conventional diagnostic angiography.. J Am Coll Cardiol.

[32] Trabold T, Buchgeister M, Küttner A, Heuschmid M, Kopp AF (2003). Estimation of radiation exposure in 16-detector row computed tomography of the heart with retrospective ECG-gating.. Fortschr Röntgenstr.

[33] Gerber TC, Stratmann BP, Kuzo RS, Kantor B, Morin RL (2005). Effect of acquisition technique on radiation dose and image quality in multidetector row computed tomography coronary angiography with submillimeter collimation.. Invest Radiol.

[34] Kondo C, Mori S, Endo M, Kusakabe K, Suzuki N (2005). Real-time volumetric imaging of human heart without electrocardiographic gating by 256-detector row computed tomography: initial experience.. J Comput Assist Tomogr.

[35] Dewey M, Hamm B (2006). Cost effectiveness of coronary angiography and calcium scoring using CT and stress MRI for diagnosis of coronary artery disease.. Eur Radiol epub.

[36] Greenland P (2006). Who is a candidate for noninvasive coronary angiography?. Ann Intern Med.

[37] Leschka S, Alkadhi H, Plass A, Desbiolles L, Grünenfelder J (2005). Accuracy of MSCT coronary angiography with 64-slice technology: first experience.. Eur Heart J.

[38] Raff GL, Gallagher MJ, O'Neill WW, Goldstein JA (2005). Diagnostic accuracy of noninvasive coronary angiography using 64-slice spiral computed tomography.. J Am Coll Cardiol.

[39] Leber AW, Knez A, von Ziegler F, Becker A, Nikolaou K (2005). Quantification of obstructive and nonobstructive coronary lesions by 64-slice computed tomography: a comparative study with quantitative coronary angiography and intravascular ultrasound.. J Am Coll Cardiol.

[40] Schuijf JD, Pundziute G, Jukema JW, Lamb HJ, van der Hoeven BL (2006). Diagnostic accuracy of 64-slice multislice computed tomography in the noninvasive evaluation of significant coronary artery disease.. Am J Cardiol.

[41] Ropers D, Rixe J, Anders K, Kuttner A, Baum U (2006). Usefulness of Multidetector Row Spiral Computed Tomography With 64-×0.6-mm Collimation and 330-ms Rotation for the Noninvasive Detection of Significant Coronary Artery Stenoses.. Am J Cardiol.

[42] Mollet NR, Cademartiri F, van Mieghem CA, Runza G, McFadden EP (2005). High-resolution spiral computed tomography coronary angiography in patients referred for diagnostic conventional coronary angiography.. Circulation.

[43] Garcia MJ, Lessick J, Hoffmann MH (2006). Accuracy of 16-row multidetector computed tomography for the assessment of coronary artery stenosis.. JAMA.

