# Supplementary material for: Patient Acceptance of Noninvasive and Invasive Coronary Angiography
Source: PLoS One. 2007 Feb 28;2(2):e246. doi: 10.1371/journal.pone.0000246 (PMC1796945; doi:10.1371/journal.pone.0000246)
Supplement: Text S1 — Patient Acceptance Questionnaire. Main Variables of Patient Acceptance. (0.09 MB PDF) [file pone.0000246.s001.pdf]

Supporting Information

Patient Acceptance Questionnaire CT/MRI/Angio Patient No.: ………………………

CT

1. Please rate the preparation and information prior to this test.

   Very good ❍ Good ❍ Barely Acceptable ❍ Poor ❍ Very poor ❍

2. Please rate your degree of concern prior to this test.

   No concern ❍ Little ❍ Moderate ❍ Intense ❍ Very intense ❍

   In case you were concerned: **Why** were you concerned? ……………………………..

3. Please rate the comfort of this test.

   Very good ❍ Good ❍ Barely Acceptable ❍ Poor ❍ Very poor ❍

4. Please rate your degree of helplessness during this test.

   No helplessness ❍ Little ❍ Moderate ❍ Intense ❍ Very intense ❍

5. Please rate your pain during and after this test on the following scale.

   No pain |————————————————| *Maximum pain*

6. Would you be willing to undergo this test again?

   Yes ❍ No ❍ Don't know ❍

7. Please rate your overall satisfaction with this test.

   Very good ❍ Good ❍ Barely Acceptable ❍ Poor ❍ Very poor ❍

MRI

1. Please rate the preparation and information prior to this test.

   Very good ❍ Good ❍ Barely Acceptable ❍ Poor ❍ Very poor ❍

2. Please rate your degree of concern prior to this test.

   No concern ❍ Little ❍ Moderate ❍ Intense ❍ Very intense ❍

   In case you were concerned: **Why** were you concerned? ……………………………..

3. Please rate the comfort of this test.

   Very good ❍ Good ❍ Barely Acceptable ❍ Poor ❍ Very poor ❍

4. Please rate your degree of helplessness during this test.

   No helplessness ❍ Little ❍ Moderate ❍ Intense ❍ Very intense ❍

5. Please rate your pain during and after this test on the following scale.

   No pain |————————————————| *Maximum pain*

6. Would you be willing to undergo this test again?

   Yes ❍ No ❍ Don't know ❍

7. Please rate your overall satisfaction with this test.

   Very good ❍ Good ❍ Barely Acceptable ❍ Poor ❍ Very poor ❍

Supporting Information

Angio

1. Please rate the preparation and information prior to this test.

    Very good ❍ Good ❍ Barely Acceptable ❍ Poor ❍ Very poor ❍

2. Please rate your degree of concern prior to this test.

    No concern ❍ Little ❍ Moderate ❍ Intense ❍ Very intense ❍

    In case you were concerned: **Why** were you concerned? ……………………………..

3. Please rate the comfort of this test.

    Very good ❍ Good ❍ Barely Acceptable ❍ Poor ❍ Very poor ❍

4. Please rate your degree of helplessness during this test.

    No helplessness ❍ Little ❍ Moderate ❍ Intense ❍ Very intense ❍

5. Please rate your pain during and after this test on the following scale.

    No pain |————————————| *Maximum pain*

6. Would you be willing to undergo this test again?

    Yes ❍ No ❍ Don't know ❍

7. Please rate your overall satisfaction with this test.

    Very good ❍ Good ❍ Barely Acceptable ❍ Poor ❍ Very poor ❍

Summary

1. Which test would you prefer for future imaging of the coronary arteries?

……..………..…………

2. Please describe other important thoughts about the three tests on the following lines and name the advantages and disadvantages of the tests using the table below.

………………………………………………………………………………………………………..
………………………………………………………………………………………………………..
………………………………………………………………………………………………………..

	**Advantages**	**Disadvantages**
CT		
MRI		
Angio		
